# Violence and its related factors among infertile women attending assisted reproductive technique unit at Al-Azhar University, Cairo

**DOI:** 10.1186/s12889-024-19433-6

**Published:** 2024-07-31

**Authors:** Doaa Sadek Ahmed, Asmaa Mohammed Abo Elela, Samar Samy Ismail, Abeer A. Almowafy, Hanaa Abou-Elyazid

**Affiliations:** 1 Community and Occupational medicine department, faculty of medicine for girls Al-Azhar University, Cairo, Egypt; 2 International Islamic Center for Population Studies and Research, Al-Azhar University, Cairo, Egypt

**Keywords:** Violence exposure, Infertile women, Husband

## Abstract

**Background:**

Violence against women is a distressing issue particularly when they are infertile. Nevertheless, many women who are infertile and exposed to violence continue their marriage and justify such choice.

**Aim:**

The current study aimed to assess the prevalence of violence against infertile women and its associated factors.

**Participants and methods:**

This cross-sectional study involved 364 Egyptian women with primary infertility; they were randomly selected from the assisted reproductive technique unit of Al-Azhar University’s International Islamic Center for Population Studies and Research. The data were collected through an interview questionnaire including the Infertile Women’s Exposure to Violence Determination Scale (IWEVDS), socio-demographic, conception, and community-related factors.

**Results:**

Moderate/high violence level was detected among 50.5% (95% CI = 45.3- 55.8%) of the studied infertile women, the mean ± SD of total score of IWEVDS was 48.27 ± 21.6. Exclusion was the most frequent type of violence among them. Binary logistic regression revealed that wives who had lower-educated husbands, lived in low-income families, had undergone prior IVF treatment, and who perceived gender inequality acceptance in society were more likely to expose to violence than others (OR = 3.76, 4.25, 2.05, and 2.08 respectively) (*P* value < 0.05).

**Conclusion and recommendations:**

Infertile women have frequent exposure to different types of violence and many factors were implicated in such condition. Despite exposure to violence, infertile women refused divorce because they had no alternative financial sources as well as they were afraid of loneliness. A community mobilization approach to control this problem through a collaboration of all stakeholders is recommended.

**Supplementary Information:**

The online version contains supplementary material available at 10.1186/s12889-024-19433-6.

## Introduction

The United Nations Declaration on the Elimination of Violence against Women since 1993 asserts that violence against women constitutes a violation of the rights and fundamental freedoms of women and impairs or nullifies their enjoyment of those rights and freedoms. Physical, psychological, and sexual violence against women and girls, whether in public or private settings, afflicts all societies and social classes, presenting significant barriers to the attainment of equality, development, and peace [1].

Violence against women is defined as “any act of gender-based violence that results in or is likely to result in, physical, sexual, or mental harm or suffering to women, including threats of such acts, coercion or arbitrary deprivation of liberty, whether occurring in public or in private life.“WHO declared that intimate partner violence and sexual violence are the most common forms of violence” [2].

Worldwide, nearly 1 in 3 (30%), of women have been subjected to physical and/or sexual violence by an intimate partner or non-partner violence or both according to prevalence data from 2000 to 2018 across 161 countries and areas. It has been discovered that women with infertility are twice more likely to be at risk of suffering from violence than women who have children [3].

Infertility constitutes a major health problem which is defined as failure to achieve a pregnancy after one year or more of regular unprotected sexual intercourse. It can be primary when a pregnancy has never been achieved or secondary when at least one prior pregnancy has been achieved [4]. Being unable to conceive can have a detrimental impact on a couple’s social life, emotional state, marital relationship, future goals, self-esteem, and body image [5]. Furthermore, the significance of fertility in culture and society cannot be overstated, especially for women, who tend to be blamed and feel more responsible for their infertility, which can lead to psychological distress [6].

Low- and middle-income countries (LMICs) have higher rates of partner violence among infertile women than the general population. Previous studies have reported past 12 months prevalence rates of 35.9% in China, 10.5–26.9% in Nigeria, 61.8% in Iran, 30.4% in Nepal, and 15–87·8% in Turkey [7], with a systematic review donated overall pooled prevalence equal to 47.2% [8]. Further studies have also been conducted on Egyptian women and revealed similar Figs. [9–12].

A variety of factors may influence the prevalence of violence among infertile women which include personal factors (such as socio-demographic and infertility factors), interpersonal factors (husband characteristics such as education, age, substance abuse, tobacco and alcohol consumption), and social factors (culture, income, and community perceptions of gender equality) [13]. Measuring these factors over time is one way to monitor progress in addressing the problem after implementing an intervention strategy [14].

The presence of traditional beliefs and practices, coupled with low income and limited education, may heighten women’s vulnerability to domestic violence. If a woman lacks access to education or economic opportunities, her ability to leave an abusive relationship may be restricted. This is particularly true if her community ostracizes her or if she lacks support from her social circle [15]. Consequently, women’s endurance of exposure to violence may be attributed to sake for their children, economic dependence, religious and familial pressures, fear of solitude, cultural constraints, and other factors [16].

Research on violence against infertile women in Egypt has predominantly focused on assessing prevalence among those attending outpatient clinics in hospitals serving a population with similar characteristics. These studies have primarily examined personal, husband-related, and conception-related factors. Therefore, the present study aims to evaluate violence and its various forms against primary infertile women at a specialized infertility center, as well as to identify potential factors contributing to the perpetration of violence, including societal influences and women justification for continuing their marriage in such abusive environments.

*Research Questions.* This study probed the following research questions:

- What’s the prevalence of violence against infertile women? Which form of violence is the most common?
- What are possible factors related to violence exposure?
- Why does infertile woman who exposed to violence refuse to get divorced or separated?

## Participants and methods

### Study design and setting

A cross-sectional study was conducted among 364 women with primary infertility for 13 months (from December 2022 to January 2024). Participants were referred for the first time to consult treatment at the assisted reproductive technique unit which is a special unit at Al-Azhar University’s International Islamic Center for Population Studies and Research. This center is considered the most popular and well-equipped infertility treatment center related to Al-Azhar University in Cairo.

### Sample size and sampling technique

The total sample size (364 women) was calculated according to the following formula; (n=[(Zα/2)^2^P(1- P)]/d^2^) [17] by considering the 95% confidence interval (Zα/2 = 1.96), a margin of error (d) of 5%, and prevalence of violence against infertile women (*P* = 36%) based on the result from a previous study [7]. The sample was recruited on three randomly selected days per week by a systematic random sampling technique from the list of patients attending the outpatient clinic of the assisted reproductive technique unit.

### Study population

The study included primary infertile Egyptian women who agreed to participate. Women receiving medical care during the research period were excluded.

The reasoning behind selecting these women as primary infertile women are more likely to experience violence compared to secondary infertile ones whose prior conception—even if it was only once—may have ensured some degree of respect and admiration from those around them. Additionally, hormonal medications that upset patients and may influence their reactions may be part of the treatment.

**Study tools** An interview semi-structured questionnaire was designed after reviewing the relevant literature. It consists of five sections and takes about 20 min to be completed. The tool was developed to collect the following data:

1. **Personal and demographic data of the couple** i.e., age, residence, education, occupation, general household income.
2. **Data related to marriage and conception** i.e., duration of marriage, duration, and causes of infertility as clinically diagnosed, and history of previous trials for assisted reproduction.
3. **Determining whether the infertile women were exposed to any form of violence** was done by using the “Infertile Women’s Exposure to Violence Determination Scale*“*(IWEVDS) which was designed to detect the risk of violence among infertile women [18]. It consists of 31 questions divided into 5 domains: Domestic violence (DV) (11 questions), Social pressure (7 questions), Punishment (6 questions), Exposure to traditional practices (4 questions), and Exclusion (3 questions). Response to these items varied from 1(never), to 5 (all the time).

The total score of violence was calculated by adding up the scores of all domains, it ranged from 31 (the minimum score) to 155 (the maximum score). Participants were classified according to quartiles of the questionnaire (mild violence: below lower quartiles, moderate violence: between the lower and upper quartiles, and high violence: above the upper quartile). Both moderate and high levels of violence were merged as having violence exposure while low levels were considered as having no such exposure.

The questionnaire was initially developed in English, and the following steps were taken for translation and cross-cultural adaptation: Two bilingual professional translators completed the forward translation into Arabic independently. Backward translation was utilized to validate the accuracy of the translation and ensure the preservation of item meanings. An expert committee consisting of two public health professionals and one research methodologist evaluated the format’s clarity and the content’s suitability. The pre-final Arabic version of the questionnaire was pilot-tested on 10% of the calculated sample size to assess the adaptability, simplicity of the questionnaire items, and questionnaire reliability.

The questionnaire’s reliability was evaluated by examining test-retest reliability and internal consistency. Test-retest reliability involved administering the questionnaire twice to the same patient two weeks apart using the same method, resulting in a strong Pearson correlation coefficient of 0.73. Internal consistency was assessed using Cronbach’s alpha coefficient, which demonstrated good reliability at 0.81.

4. **Possible factors associated with violence**: [19]
  - **Factors related to spouse behavior** (e.g., alcohol consumption, drug addiction, suffering from any psychological problem, history of violence exposure during early life).
  - **Factors related to society perspective toward violence** include community acceptance of violence phenomenon and media encouragement of violence.
5. **Reasons for refusal of divorce among women exposed to violence** as fear of loneliness, lack of social or financial support, or no acceptance of divorce stigma.

### Operational definitions of violence forms

- Exclusion: unjust attribution of blame, exclusion from decision-making and constant comparison to fertile women.
- Punishment: pressure for sexual intercourse, nicknaming, being subjected to exhausting house work, …. etc.
- Traditional practice: forcing infertile women to eat some type of food believed to facilitate conception, exposure to inquisitive questions about having a child making them tell a lie or give an evasive answer.
- Social pressure: any social difficulties such as stigma, isolation, humiliation, gossip, being made to feel guilty and disabled by the community.

### Data management and statistical analysis

Data were analyzed using the IBM Statistical Package for Social Science version 20. Numerical data were expressed as mean ± SD or median and Interquartile range according to data distribution while qualitative data were expressed as frequency and percentage. Comparisons between variables of qualitative data were performed using the chi-square test while quantitative variables were compared by independent-samples t-test and Mann- Whitney U test instead when data were non-normally distributed. Logistic regression was performed to determine associated factors of exposure to violence among participants. Probabilities (p-values) of less than 0.05 are considered significant at 95% confidence level.

## Results

The current study was conducted on a total sample of 364 infertile women. The mean age ± SD of infertile women and their husbands was 30.65 ± 5.55 and 35.59 ± 5.67 years respectively. Furthermore, 77.2% & 84.3% of women and their husbands respectively had higher educational levels. Regarding working status, nearly three-quarters (73.9%) of the studied women were housewives, while 93.2% of their husbands had work. A substantial percentage of these women (71.2%) resided in urban areas. Additionally, 70.4% of families had income that just met their basic requirements, and the mean duration of their marriage was 5.75 ± 3.7 years. According to the history of infertility, most of the studied women (89.1%) suffered from infertility for a duration ranging from one to ten years, with unexplained causes among 56.9% of them. Moreover, 60.2% of participants didn’t undergo any previous assisted reproductive techniques as shown in Table (S1, S2) in the supplementary file.

**Fig. 1:**
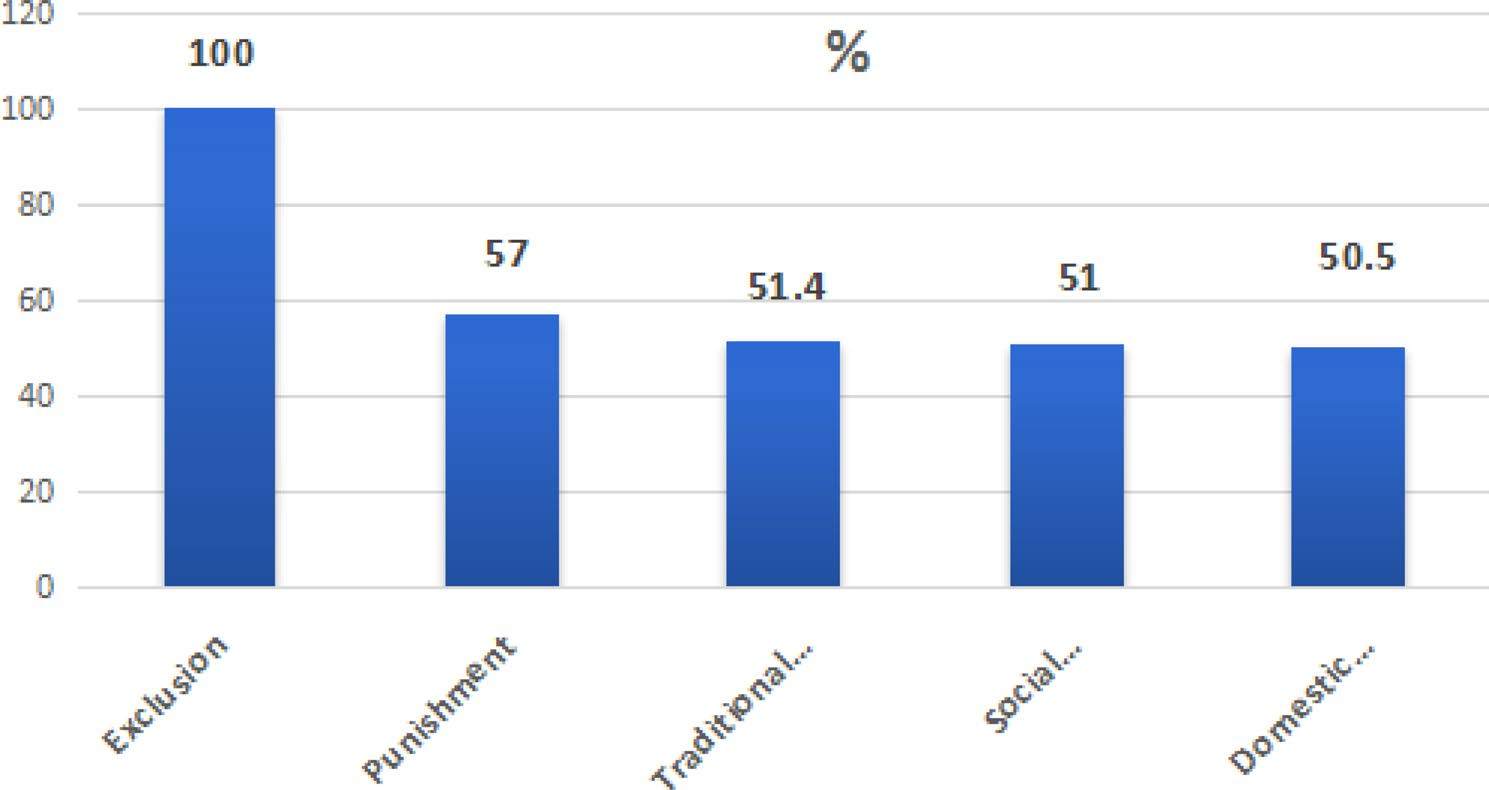
Distribution of different types of violence among the studied women

As regards violence exposure, the (IWEVDS) total scores ranged from 31 to 139, with mean ± SD equals 48.27 ± 21.6. Exposure to moderate/ high violence levels was detected among 50.5% (95% CI = 45.3- 55.8%) of the participants in which all of them were exposed to exclusion form, while 57.0% of them were exposed to punishment and nearly half of them were exposed to traditional practice, social pressure, and domestic forms (51.4%, 51.0%, and 50.5% respectively) as in Fig. 1.

Regarding factors affecting violence exposure, Table 1 clarifies that older and not working husbands, low educational level of both partners and low family income are associated with moderate/ high violence exposure with statistically significant differences (P value < 0.05).

**Table 1:** Socio-demographic characteristics among the studied sample according to violence exposure

Variables	Moderate / High violence exposure (184)	Low violence exposure (180)	Significant Value
**Age of wife: Mean (SD)**	31 (5.7)	30.3 (5.4)	*p*. value = 0.23**
**Age of husband: Mean (SD)**	36.3 (5.8)	34.8 (5.4)	*p*. value = 0.02**
**Wife educational level: No (%)**			
Low	57 (31.0)	26 (14.4)	*p*. value = 0.00*
High	127 (69.0)	154(85.6)	
**Husband educational level: No (%)**			
Low	44 (23.9)	13 (7.2)	*p*. value = 0.00*
High	140 (76.1)	167(2.8)	
**Wife working status: No (%)**			
Not working (Housewife)	140 (76.1)	129 (71.7)	p. value = 0.33*
Working	44 (23.9)	51 (28.3)	
**Husband working status: No (%)**			
Not working	17 (9.2)	4 (2.2)	*p*. value = 0.000*
Working	167 (90.8)	176 (97.8)	
**Residence: No. (%)**			
Rural	54 (29.3)	51 (28.3)	*p*. value = 0.83*
Urban	130 (70.7)	129 (71.7)	
**Family income: No. (%)**			
Inadequate	27 (14.7)	20 (11.1)	*p*. value = 0.000*
Just meet basic requirements	142 (77.1)	114 (63.3)	
Meat basic requirements	15 (8.2)	44 (24.4)	
Able to save and invest money	0 (0.0)	2 (1.2)	
**Duration of marriage**:			
Median (IQR)	5 (3–7)	5 (3–7)	*p*. value = 0.65***

* Pearson Chi -Square (*X*^*2*^*)* test, ** Independent sample *t* test, *** Mann –Whitney U

Additionally, moderate/ high violence level was associated with infertility attributed to the wife or unexplained cause and previous attempts of IVF treatment with statistically significant difference (P value < 0.05) as shown in Table 2.

**Table 2:** Infertility history of the studied women in relation to violence exposure

Variables	Moderate / High Violence exposure (184)	Low Violence exposure (180)	Significant value
**No. (%)**			
**Duration of infertility /years :**			
< 5	91 (49.5)	85 (47.2)	*p*. value = 0.54*
5–10	69 (37.5)	79 (43.9)	
10–15	20 (10.9)	14 (7.8)	
> 15	4 (2.1)	2 (1.1)	
**Responsibility for infertility**:			
Husband	23 (12.5)	27 (15.0)	*p*. value = 0.004*
Wife	23 (12.5)	12 (6.7)	
Both of them	47 (25.5)	25 (13.9)	
Un- explained	91(49.5)	116 (64.4)	
Previous IVF**:			
No	100 (54.3)	119 (66.1)	*p*. value ***=*** 0.022*
Yes	84 (45.7)	61 (33.9)	

* Pearson Chi - Square (*X*^*2*^*)* test. **In vitro fertilization

Moreover, husband-related factors associated with moderate/ high violence were multiple sexual relations outside marriage, lack of alcohol intake, and those with no history of parent separation or divorce during childhood with statistically significant differences (P value < 0.05) as in Table 3. Additionally, by comparing alcoholic and non alcoholic husbands as shown in Table (S3) in the supplementary file, there was a difference between both groups regarding level of education and income.

**Table 3:** Husband-related factors of violence among the studied women

Variables	Moderate / high violence exposure (184)	Low violence exposure (180)	Significant value
**No. (%)**			
**Alcohol intake**:			
Yes	13 (7.1)	24 (13.3)	***p***. **value** = 0.04*
No	171 (92.9)	156 (86.7)	
**Drug addiction**:			
Yes	20 (10.9)	22 (12.2)	*p*. value = 0.686*
No	164 (89.1)	158 (87.8)	
**Childhood experience of violence between his parents**:			
Yes	39 (21.2)	39 (21.7)	*p*. value = 0.913*
No	145 (78.8)	141 (78.3)	
**Separation/divorce of his parents when he was child**:			
Yes	17 (9.2)	29 (16.1)	***p***. value = 0.04*
No	157 (90.8)	151 (83.9)	
**Violence exposure during childhood**:			
Yes	39 (21.2)	30 (16.7)	*p*. value = 0.27*
No	145 (78.8	150 (83.3)	
**History of mental illness/ personality disorders**:			
Yes	33 (17.9)	29 (16.1)	*p*. value = 0.644*
No	151(82.1)	151 (83.9)	
**Multiple sexual relation outside of marriage**:			
Yes	42 (22.8)	26 (14.4)	***p***. **value** = 0.04*
No	142 (77.2)	154 (85.6)	
**Has other wives**:			
Yes	38 (20.7)	31 (17.2)	*p*. value = 0.404*
No	146 (79.3)	149 (82.8)	
**Living in expanded family dwelling** :			
Yes	115 (62.5)	117 (65.0)	*p*. value = 0.620*
No	69 (37.5)	63 (35.0)	

* Pearson Chi -Square (*X*^*2*^*)* test

**Fig. 2:**
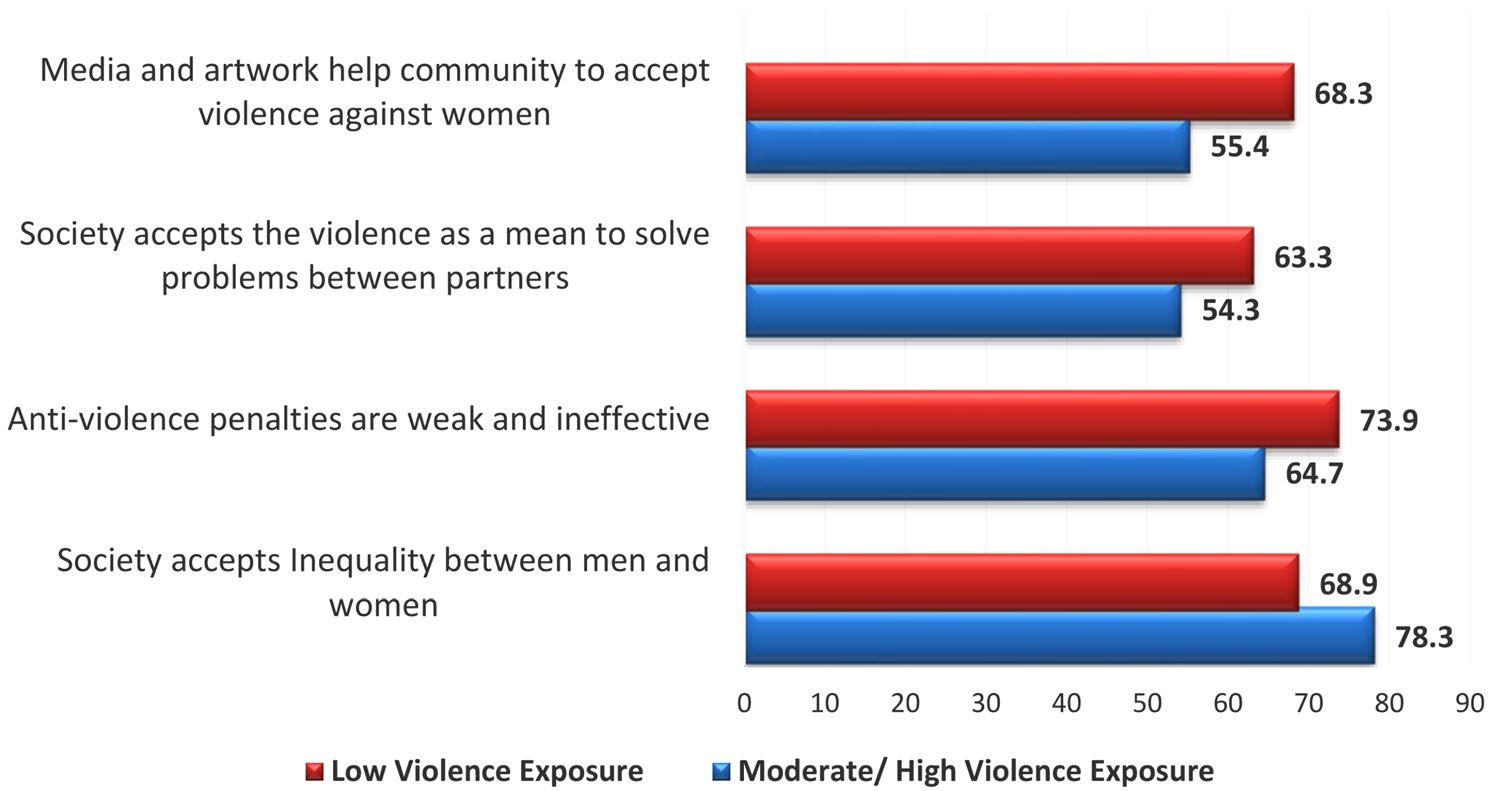
Society -related factors of violence as perceived by the studied women

Society- related factors of violence as perceived by women were recorded as follow: “societal acceptance of gender inequality” was perceived as a contributing factor of violence among moderate/ high violence exposure group more than their counterparts. On the other hand, “weak and ineffective anti-violence penalties”, “societal acceptance of violence as a mean to solve problems between partners”, and “media products help community acceptance to violence against women” were recorded more among low levels of violence exposure group than those with moderate/ high level Fig. 2.

Logistic regression analysis presented in Table 4 reveals that a husband with lower educational attainment was more inclined to perpetrate violence against his infertile spouse (OR = 3.76). Furthermore, infertile women residing in low-income households, those who had undergone previous IVF treatment, those who perceived societal acceptance of gender inequality, and those who perceived a lack of media influence in discouraging violence against women were more susceptible to experience violence compared to others (OR 4.25, 2.05, 2.08, and 2.56 respectively) (P value < 0.05).

**Table 4:** Binary logistic regression for detecting factors associated with moderate/ high violence among infertile women

Variables	Univariate analysis		Multivariate analysis	
	OR	95% C.I.	OR	95% C.I.
Family income (high/low)	3.9	2.07–7.22	**4.25**	2.15–8.37
Husband educational level (high/low)	4.03	2.09–7.79	**3.76**	1.89–7.48
Role of media in violence acceptance against women as perceived by women (yes/ no)	1.73	1.13–2.66	**2.56**	1.17–3.69
Societal acceptance of gender inequality as perceived by women (no/ yes)	1.62	1.01–2.60	**2.08**	1.17–3.69
Previous IVF treatment (no/ yes)	1.63	0.39–0.93	**2.05**	1.28–3.29

*Statistically significant predictor (*p*. value < 0.05)

**Fig. 3:**
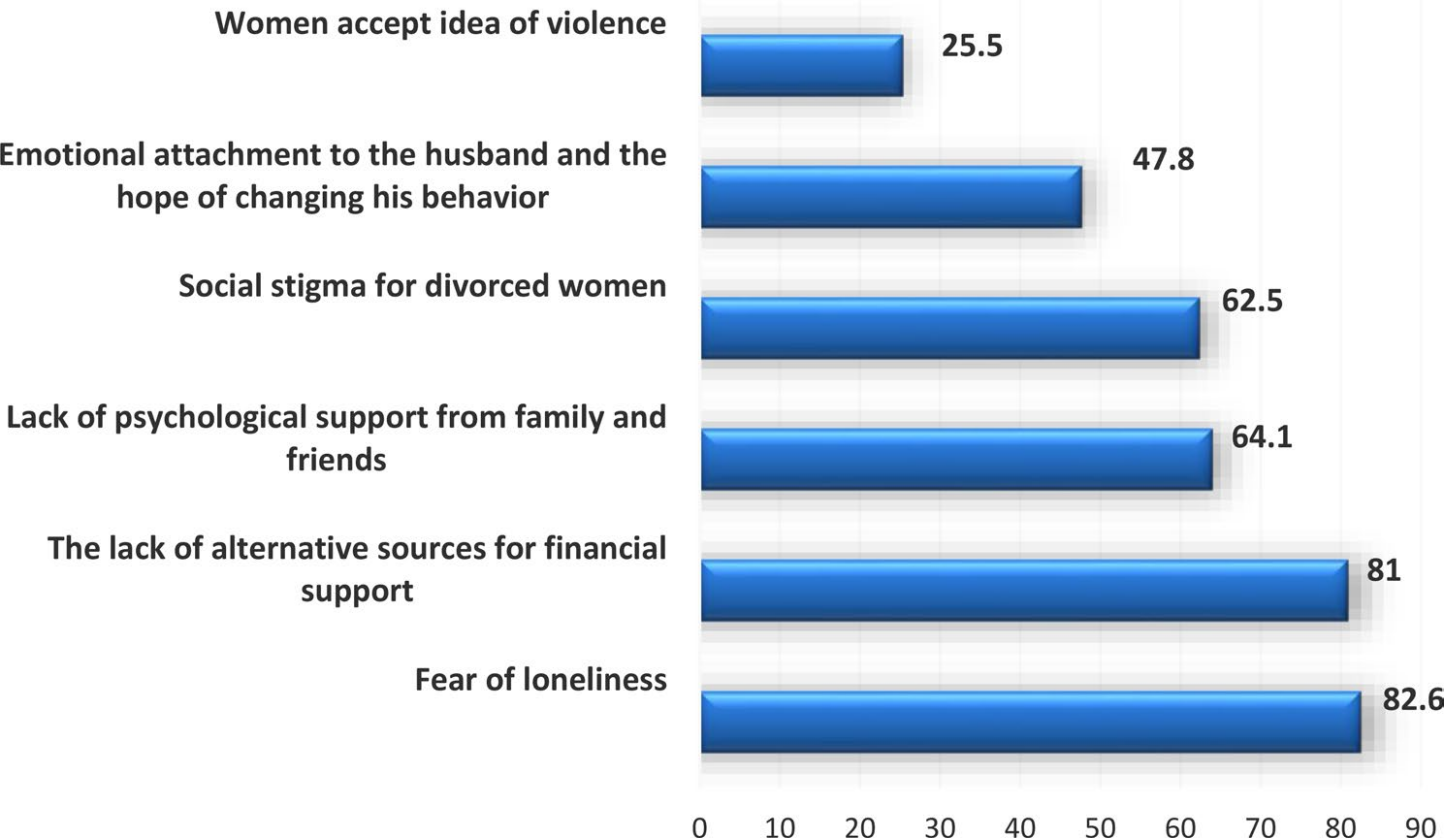
Causes of refusing divorce among infertile women exposed to moderate/high violence

Many reasons justifying the refusal of divorce were recorded by infertile women exposed to violence as fear of loneliness, lack of alternative financial and social support from family and friends, Social stigma for divorced women, emotional attachment to husband with hope to change his behavior, and their acceptance of the idea of violence Fig. 3.

## Discussion

Infertile women in developing countries experience various forms of violence, including physical, emotional, sexual, and psychological abuse in which partners, family members, or even society may be the perpetrators. So, the present study was designed firstly to determine the prevalence of violence against primary infertile women and to identify its possible factors. It was found that 50.5% of our participants were exposed to moderate/high violence levels, and the mean total score of IWEVDS was 48.27 ± 21.6, in which exclusion form of violence was the most frequent one mentioned by all the studied women, followed by punishment (57%), and nearly half of them were exposed to traditional practice, social pressure, and domestic violence.

These findings corroborate a great deal of previous work that is interested in this issue. In Egypt, Elkateeb found that 58.2% of infertile women were often exposed to violence with verbal form being the most common and sexual violence being the least common, and only 3% were vulnerable to all types of domestic violence [20]. Then, Lotfy et al. used the IWEVDS among infertile women and revealed a higher total mean score of 73 ± 17, with the dominant types being domestic, punishment, and exposure to traditional practices domains [10]. Also, Mohamed &Ezz Eldin used the same scale and showed that the highest proportion of infertile women subjected to more than one type of violence scored highly for domestic violence, punishment, social pressure, exposure to traditional practices, and social exclusion [12]. In addition, Ghoneim et al. mentioned primary infertility as a significant contributing factor to infertile women’s exposure to violence [11]. While Hassan et al. compared upper and lower Egyptian infertile women regarding violence and discovered that they experienced and were exposed to all forms of domestic one [21].

Furthermore, a systematic review and meta-analysis conducted by Sharifi et al. using data from 15 studies revealed that the prevalence of violence against infertile women ranged between 14.9% and 88.9% with an overall pooled prevalence of 47.2%. A comparison of domestic violence between fertile and infertile women was reported in five studies in which four studies demonstrated that spousal violence increased significantly in response to infertility. Among the most prevalent forms of violence were psychological and emotional abuse [8].

Many factors may influence the problem of violence against infertile women, which can be categorized into individual, interpersonal, and societal factors as well as other factors related to infertility. We discovered that high levels of violence exposure among infertile women were significantly linked to low educational attainment of partners, not working husbands, and low-income families.

These results are in line with Mansour & Mohdy who found that women with low education and low family income were more likely to suffer from severe violence compared to the other group of women and that their husbands also followed this pattern [9]. Similar results were observed by other studies [22–24].

While addressing husband-related factors of violence, the current study found that multiple sexual relations outside marriage were significantly associated with high violence exposure, which is consistent with a finding of Rahaman et al. that revealed women in polygyny unions faced significantly higher spousal violence than monogamy unions [25]. Another unexpected factor was that husbands who reported drinking alcohol were less likely to be predators. Conversely, Aduloju et al. disclosed that infertile women whose spouses had a habit of smoking and drinking alcohol significantly experienced violence [26]. Although the reason for this discrepancy is unclear, alcohol consumption may have detached the husband’s attention from the infertility problem. Additionally, nearly three-fourths of participants who drank alcohol in our study had high levels of education, which could account for their wives’ lack of violence.

For conception-related factors that contribute to violence, we found that infertility reasoned by wives, and those who were previously managed by IVF were significantly associated with high violence levels with no significant difference as regards the duration of marriage or infertility. In accordance, Akyuz et al. and Taebi et al. revealed that women with female or unknown causes of infertility are more likely to experience violence which could be due to the concept of male dominance and the likelihood of blaming wives solely about issues related to sexual or reproductive health in some cultures [27, 28]. Conflict outcomes were previewed by other studies., as Mansour& Mohdy observed that women who experienced more than one trial of traditional treatment had significantly severe intimate partner violence (IPV), but in contrast, they demonstrated that the longer the duration of marriage, the greater the severity of IPV [9]. Also, Omidi et al. found a direct relationship between the total score of violence and the duration of the marriage duration of infertility, and the duration of infertility treatment [23], while Akyuz et al. noted that the total score of marital violence was higher in women who had been trying to have children for more than 6 years and those who had been treated for infertility for more than 3 years [29].

Prior research revealed very little about societal factors. Even while there may not be any particular cultural elements that directly lead to violence, stigmatization can still be facilitated by societal attitudes and misconceptions. According to the results of the current study, women who had experienced high levels of violence were significantly more likely to believe that men and women are treated unequally in society, but fewer of them thought that the media played a positive role in the public’s acceptance of violence against women.

These findings are in agreement with Kearns et al. who found that gender inequality represents an important societal-level factor associated with violence among women [30]. In addition, Abramsky et al. demonstrated that the perception of gender inequality is one of the elements fueling this violence; it may indirectly affect the reactions of service providers, as well as friends, neighbors, and coworkers of women exposed to violence. Attitudes have the power to affect both the victims of violence and their perpetrators [31]. A possible explanation for the studied women’s perception might be due to the recent establishment of various initiatives in Egypt to address anti-women violence which was extensively spared by the media.

Although violence is never acceptable by anyone and is always regarded as a violation of human rights, some reasons may affect the decision of violent women to stay in their marriage, the current study spotted some of these reasons that may widely spread in our community in which fear of loneliness, lack of financial and social support from family and friends, and social stigma for divorced women were the most frequent reasons mentioned by violent women, which is inconsistent with Lotfy et al. who found by multivariable linear regression analysis that the best-fitting predictors for this high violence scale were divorce threatens and fear from husband [10].

## Limitations

The current study was subjected to some limitations due to relatively small sample of included population. Also it doesn’t assess the consequences of violence on the women that may be necessary for further research.

## Conclusion

Infertile women have frequent exposure to different types of violence with a predominance of exclusion, punishment, and traditional practice types. Furthermore, high violence exposure had a significant association with women of: low-educational level, non-alcoholic husband, husband with multiple sexual relations outside marriage, low-income families. Additionally, wives who were exposed to previous IVF treatment, perceived societal acceptance of gender inequality, and perceived no roles of media in community acceptance of violence against women, are more likely to expose to violence than others. Despite being victims of violence, infertile women refused divorce because they had no alternative financial sources as well as they were afraid of loneliness.

## Recommendations

In the light of our findings, a community mobilization approach is required to reduce the burden of the problem of violence against infertile women and enhance their well-being through a collaboration of all stakeholders. *At the individual level*, the government should empower infertile women psychologically and economically through the eradication of illiteracy, providing financial support for the establishment of small projects, and offering digital applications for their support and reassurance when needed. *At the interpersonal level*, it’s necessary to train healthcare providers effectively, at the primary level, for couple counseling to eliminate marital apathy and enhance emotional communication between partners helping them to cope with the stressors and conflicts associated with infertility and empower them to interact with the community stigma-free. Fortunately, it has been started through Safe Women Clinics which were established in various primary health care units in 2022 under the supervision of the Ministry of Population and Health in cooperation with the National Council for Women, the United Nations Fund, and the General Secretariat for Mental Health. *At the society level*, it’s crucial to raise community awareness for gender equality and correction of the misconception that woman solely is the cause of infertility. Finally, evaluation of intervention strategies and additional research on the consequences of violence against infertile women should be prioritized.

### Electronic supplementary material

Below is the link to the electronic supplementary material.

Supplementary Material 1

Supplementary Material 2

## Data Availability

Data is provided within the manuscript or supplementary information files.
