# Supplementary material for: Violence and its related factors among infertile women attending assisted reproductive technique unit at Al-Azhar University, Cairo
Source: BMC Public Health. 2024 Jul 31;24:2063. doi: 10.1186/s12889-024-19433-6 (PMC11293121; doi:10.1186/s12889-024-19433-6)
Supplement: Supplementary file 1 — Supplementary Material 1 [file 12889_2024_19433_MOESM1_ESM.docx]

**Table (S1): -Socio-demographic characteristics of the studied women and their husbands.**

| **Items** | **Total No.= (364)** |
| --- | --- |
| **Age of wife**: -   - Mean ± SD - (min. - max.)   **Age of husband: -**   - Mean ± SD - (min. - max.) | 30.65 ± 5.55  (20 – 49)  35.59 ± 5.67  (23 – 52) |
| **Wife Educational level: -**   - Low educational level - High educational level     **Husband Educational level: -**   - Low educational level - High educational level | **No. (%)** |
|  | 83 (22.8)  281 (77.2)  57 (15.7)  307 (84.3) |
| **Occupation of wife: -**   - Housewife - Employee   **Occupation of husband: -**   - Not working - Had a work (Governorate employee/ Free business | 269 (73.9)  95 (26.1)  21 (5.8)  343 (94.2) |
| **Residence: -**   - Rural - Urban | 105 (28.8)  259 (71.2) |
| **Family Income: -**   - Inadequate - Just meet basic requirements - Meet basic requirements & emergencies - Able to save and invest money | 47 (12.9)  256 (70.4)  59 (16.2)  2 (0.5) |
| **Duration of marriage**   - (mean ± SD) - (Min. – Maxi.) | 5.75 ± 3.65  (1-23) |

**Table (S2): -Infertility History among studied women.**

| **Items** | **No. (%)** |
| --- | --- |
| **Duration of infertility \ years**   - < 5 - 5 – 10 - 10 -15 - >15 | 176 (48.4)  148 (40.7)  34 (9.3)  6 (4.4) |
| **Responsible for infertility: -**   - Husband - Wife - Both of them - Un- explained | 50 (13.7)  35 (9.6)  72 (19.8)  207 (56.9) |
| **Previous IVF: -**   - No - Yes | 219 (60.2)  145 (39.8) |

**Table (S3): - Husband Alcohol Intake and Its Relation to His Educational Level and Family Income**

| **Variables** | **Husband drinking alcohol beverages** | | **Significant Value** |
| --- | --- | --- | --- |
|  | **Yes (37)** | **No (327)** |  |
| **Family income:**   - Low - High | **No (%)** | **No (%)** | **p. value** = **0.051***  OR= 3.9  C.I: (0.06-1.10) |
|  | 35 (94.6)  2 (5.4) | 268 (82.0)  59 (18.0) |  |
| **Husband educational level:**   - Low educational level - High educational level | 8 (21.6)  29 (78.4) | 49 (15.0)  278 (85.0) | p. value = 0.29*  OR= 1.6  C.I: (0.28-1.48) |

* Pearson Chi -Square (*X^2^)* test., OR: - odds ratio C.I: - confidence interval.
