# Supplementary material for: Violence and its related factors among infertile women attending assisted reproductive technique unit at Al-Azhar University, Cairo
Source: BMC Public Health. 2024 Jul 31;24:2063. doi: 10.1186/s12889-024-19433-6 (PMC11293121; doi:10.1186/s12889-024-19433-6)
Supplement: Supplementary file 2 — Supplementary Material 2 [file 12889_2024_19433_MOESM2_ESM.pdf]

Survey questionnaire about forms and factors related to violence against primary infertile married women.

**Section 1: Demographic characteristics of the family**	
**Wife's age:**	…………………………….. years
**Place of residence:**	1. Rural/village<br>2. Urban/city
**Wife's educational level:**	1. Illiterate<br>2. Basic primary educational level<br>3. Intermediate or upper-intermediate education<br>4. University education - postgraduate level
**Wife's job status:**	1. Housewife<br>2. Government employee<br>1. Freelance work
**Husband's age:**	…………………………….. years
**Husband's educational level:**	1. Illiterate<br>2. Basic primary educational level<br>3. Intermediate or Intermediate or upper-intermediate education<br>4. University education - postgraduate level
**Husband's job status:**	3. Unemployed<br>4. Government employee<br>5. Freelance work
**Family income:**	1. Not enough<br>2. Enough for basic requirements<br>3. Enough for basic and urgent requirements<br>4. Enough and surplus for savings and investment

**Section 2: History of infertility**	
**Marriage duration:**	…………………………….. years
**Duration of infertility:**	..……………………………years
**Medical reasons for infertility?**<br>May be related to……….	1. Husband<br>2. Wife<br>3. Both together<br>4. Unexplained infertility
**Have you ever subjected to any Assisted Reproductive techniques such as artificial insemination or intracytoplasmic sperm injection (ICSI)?**<br>If the answer is yes: How many times have you tried?	1. Yes<br>2. No<br>…………………………………

Section 3: Forms of violence "Infertile Women’s Exposure to Violence Determination Scale"(IWEVDS)*					
**In the ls the last year, did you expose to the following?**	**All the time**	**Often**	**Some Times**	**Rarely**	**Never**
**Domestic violence domain**					
1. My partner threatens me with divorce because I am childless.					
2. My partner abstains from kissing and touching me because I am childless.					
3. My family abstains from giving me property rights because my disability hinders the reproduction of my family.					
4. My partner thinks on getting married to a fertile woman since I can’t have children.					
5. My partner abstains from having sex with me because I am childless.					
6. I am exposed to kicks, fists, and slaps because I am childless.					
7. My partner abstains from visiting his family together with me because I am childless.					
8. My partner does not say words of love to me because I am childless.					
9. My partner says humiliating words to me concerning my sexual performance because I am childless (incapable, frigid etc.)					
10. I sometimes get humiliated in front of others because I am childless.					
11. Despite my unwillingness, I get insisting requests to visit some relatives who have children.					
**Social pressure domain**					
1. I am excluded by people around me because I am childless.					
2. I am not greeted because I am childless.					
3. I am pointed out as “the childless woman”					
4. People gossip about my childlessness.					
5. People around me consider me as disabled and guilty.					
6. Because of my inability to reproduce, comments concerning my womanhood, which saddens me, are made.					
7. People around me blame me all the time because I am childless.					
**Punishment domain**					

1. I am exposed to heavy punishment such as tough housework because I am childless.					
2. Any kind of failure that I have is associated with my disability to bear a child.					
3. People give me nicknames related to my disability to bear (infertile, castrated, unproductive etc.)					
4. People sometimes do not invite me to family reunions that include children.					
5. Despite my unwillingness, my partner insists on having sexual intercourse in order to conceive all the time.					
6. Even though I am not the reason for the infertility, I am under pressure to tell other people that the disability belongs to me.					
**Exposure to traditional practices domain**					
1. I am exposed to curious questions such as "When will you give a birth?"					
2. In order not to be exposed to curious questions, I have to tell lies or give evasive answers.					
3. Despite my unwillingness, I am forced to eat some food which is believed to facilitate conception (honey, hazelnuts, restoratives, ram's testicles)					
4. Despite my unwillingness, I am exposed to several traditional practices which are thought facilitate conception (imam prays for a couple in order to break a spell etc.)					
**Exclusion domain**					
1. I am compared to fertile women all the time.					
2. I am held responsible for/being accused of any random misfortune in life because I am childless.					
3. I am not allowed in decision-making mechanisms in my family.					

**Section 3: Factors of violence related to husband, relationship, and society****	
**Does your spouse drink alcohol?**	1.yes<br>2.No<br>3. Don't know
**Does your spouse abuse drugs?**	1.yes<br>2.No<br>3. Don't know

**Was the husband exposed to violence between his parents?**	1.yes<br>2.No<br>3. Don’t know
**Did spouse's parents get divorced or separated when he was a child?**	1.yes<br>2.No
**Did husband experience any physical or sexual, as a child?**	1.yes<br>2.No<br>3. Don’t know
**Does your spouse have any psychological or mental health issues (i.e., Personality disorders)?**	1.yes<br>2.No<br>3. Don’t know
**Does husband have multiple partners outside marriage?**	1.yes<br>2.No<br>3. Don’t know
**Does husband have other wives?**	1.yes<br>2.No<br>3. Don’t know
**Are you living in separate house (away from family)?**	1.yes<br>2.No
**In your opinion………...**	
**Are there prevailing gender-inequitable social norms in your society (especially those that link notions of manhood to dominance and aggression)?**	1. Agree<br>2. Neutral<br>**3.** Disagree
**Are community sanctions against intimate partner violence weak and inactive?**	1. Agree<br>2. Neutral<br>3. Disagree
**Is there broad social acceptance of violence to resolve couple conflict?**	1. Agree<br>2. Neutral<br>**3.** Disagree
**Does the media and artwork help to foster acceptance of the idea of violence against women?**	1. Agree<br>2. Neutral<br>**3.** Disagree

**Section 5: Why don’t women leave violent partners? ****		
**Fear of loneliness**	1.Yes	2. No
**Lack of alternative means of economic support;**	1.Yes	2. No
**Lack of support from family and friends**	1.Yes	2. No
**Stigma among women who have divorced**	1.Yes	2. No
**Emotional attachment and the hope that the partner will change**	1.Yes	2. No

**Women acceptance of the concept of being violated**	1.Yes 2. No

References:

*Onat G. Development of a scale for determining violence against infertile women: A scale development study. Reprod Health. 2014;11:1–8.

**World Health Organization & Pan American Health Organization. (2012). Understanding and addressing violence against women : intimate partner violence. World Health Organization. https://apps.who.int/iris/handle/10665/77432.
Accessed 5 January 2024.
